# Parental Recognition of Bullying and Associated Factors Among Children After the Fukushima Nuclear Disaster: A 3-Year Follow-Up Study From the Fukushima Health Management Survey

**DOI:** 10.3389/fpsyt.2019.00283

**Published:** 2019-05-03

**Authors:** Misari Oe, Masaharu Maeda, Tetsuya Ohira, Shuntaro Itagaki, Mayumi Harigane, Yuriko Suzuki, Hirooki Yabe, Seiji Yasumura, Kenji Kamiya, Hitoshi Ohto

**Affiliations:** ^1^Department of Neuropsychiatry, Kurume University School of Medicine, Kurume, Japan; ^2^Radiation Medical Science Center for the Fukushima Health Management Survey, Fukushima Medical University, Fukushima, Japan; ^3^Department of Disaster Psychiatry, School of Medicine, Fukushima Medical University, Fukushima, Japan; ^4^Department of Epidemiology, School of Medicine, Fukushima Medical University, Fukushima, Japan; ^5^Department of Neuropsychiatry, School of Medicine, Fukushima Medical University, Fukushima, Japan; ^6^Department of Adult Mental Health, National Institute of Mental Health, National Center of Neurology and Psychiatry, Kodaira, Japan; ^7^Department of Public Health, School of Medicine, Fukushima Medical University, Fukushima, Japan

**Keywords:** bullying victimization, nuclear disaster, child and adolescent psychiatry, relocation, disaster mental health

## Abstract

This study examined parental recognition of bullying victimization and associated factors among evacuated children after the 2011 Fukushima Daiichi Nuclear Power Plant accident, using a 3-year follow-up data (wave 1: January 2012; wave 2: January 2013; wave 3: February 2014). The sample included the caregivers of 2,616 children in the first–sixth grades of elementary school, who lived in one of the 13 municipalities that were the target areas of the Mental Health and Lifestyle Survey, conducted as part of the Fukushima Mental Health Management Survey. Across 3 years, around 80% of caregivers responded “not true,” 15% responded “somewhat true,” and 5% responded “certainly true” in response to a question about bullying victimization of their children. Being male was significantly associated with the parental recognition of bullying victimization at wave 1 and wave 3. At wave 1, experiencing the nuclear plant explosion was significantly associated with parental recognition of bullying victimization. Moreover, age at wave 3 was negatively associated with parental recognition of bullying victimization. Our findings will be helpful for establishing community- and school-based mental health care for children, parents, and teachers.

## Introduction

The Fukushima Daiichi Nuclear Power Plant (FDNPP) disaster occurred on March, 11, 2011, after the Great East Japan Earthquake. An earthquake of magnitude 9.0 in the Tohoku area of Japan triggered a huge tsunami that reached heights of up to 40 m. In the Soma area near the FDNPP, the tsunami had a height of 9.3 m, and 1,817 people were presumed dead (1). The tsunami caused a total loss of electricity, several explosions at FDNPP buildings, and widespread diffusion of radioactive materials. A nuclear emergency was declared for the first time in Japan, and residents within 20 km of the FDNPP were evacuated. Among the approximately 2 million people living in Fukushima Prefecture at the time of the disaster, more than 160,000 people were evacuated as of May 2012. As of 5 years after the Fukushima disaster, approximately 11,000 school-aged children were relocated along with their family members (2). The Fukushima disaster was regarded as a complex of different types of disasters: earthquake, tsunami, and nuclear disaster (3, 4).

The local government of Fukushima Prefecture and Fukushima Medical University started a large cohort study called the Fukushima Health Management Survey to investigate the effects of long-term low-dose radiation exposure caused by the accident (5). The study consists of several surveys, including thyroid ultrasound examinations for all children in Fukushima aged 18 years or younger at the time of the disaster, a comprehensive health check for all residents from the evacuation zones, an assessment of the mental health and lifestyle factors of all residents from the evacuation zones, and records of all pregnancies and births among all women in the prefecture who were pregnant on March 11, 2011. A survey of mental health and lifestyle factors has been conducted annually.

Nuclear disasters are typically regarded as an “invisible” disaster. Because it is difficult to estimate the health impacts and risk associated with a nuclear disaster, the relevance of psychological consequences after a disaster may increase in the long term. Importantly, researchers have reported that the biggest impact of the Chernobyl disaster throughout the years has been on mental health (6). Thus, the concerns of parents regarding the health status of their children represent one of the most important issues involved in the aftermath of nuclear accidents. Previous studies of the Fukushima disaster reported that the mental health impacts continued for years after the disaster (7), with a difference in the impacts for evacuees and nonevacuees. Thus, the situations of the evacuees after a nuclear disaster in the long term could be considered as the “daily living in the post-disaster context.”

In the current study, we focused on bullying among children after the Fukushima disaster. A survey by the Ministry of Education, Culture, Sports, Science, and Technology of Japan found 204 cases of bullying victimization involving children who evacuated Fukushima after the nuclear disaster (2, 8). Examples of disaster-related bullying included being nicknamed “radiation,” being told to go back to Fukushima, or being accused of causing the plant explosion. One previous study reported that the prevalence of bullying victimization among children evacuated from the Fukushima Prefecture was 1.09% in 2016 (2). However, the Ministry of Education, Culture, Sports, Science, and Technology of Japan reported that the number of school bullying cases in the country under a nondisaster setting was 323,808 in 2016, with a prevalence of 2.39% in 2015 (2). According to an international survey, the proportion of students bullied under a nondisaster setting varies from 6.3% to 41.4% (9). In a study of 2,630 Japanese junior high school children, the prevalence of being bullied under a nondisaster setting was 18.6% in boys and 19.9% in girls (10). Thus, these results indicate a higher prevalence of bullying than reports by the Japanese government.

A small number of previous studies have examined bullying and bullying victimization among children after large-scale disasters. Terranova et al. (11) reported that youth from an area affected by Hurricane Katrina showed significant increases in relational and overt bullying after the hurricane (11). Although the results did not reveal a significant increase in relational peer victimization, there was a significant decrease in the comparison group over a similar period (11). In addition, the study revealed that being male and being the target of relational victimization before the hurricane predicted higher posthurricane relational victimization (11). Another study of a representative sample of 2,030 children in the United States aged 2–17 years revealed that disaster exposure was associated with some forms of victimization including peer victimization (12).

In the current study, using data from the Fukushima Health Management Survey (5), we examined the prevalence of bullying victimization and associated factors among children who experienced the Fukushima disaster, to elucidate the difference between the prevalence reported in previous study results and the reports by the Ministry of Education, Culture, Sports, Science, and Technology of Japan. Because the data regarding children were obtained from their caregivers (e.g., parents, grandparents), we examined parental recognition of bullying victimization. In addition, using a 3-year follow-up dataset, we investigated changes in the prevalence of bullying victimization over time.

We hypothesized that the proportion of bullying victimization might be associated with sociodemographic factors, including sex and age, and with disaster-related factors, such as the number of disaster types experienced, namely, earthquake, tsunami, and nuclear plant explosion, and the place of residence. With respect to sex, an international comparative study under nondisaster settings showed a higher proportion of boys being bullied than girls in most of the 28 countries studied (9). However, in a Japanese study, the prevalence of being bullied under a nondisaster setting was higher among girls than boys (10). We hypothesized that children whose current place of living was outside of the Fukushima Prefecture would have a higher proportion of bullying victimization, based on a previous study showing that out-of-prefecture evacuee children were at a greater risk of more severe emotional symptoms compared with children who remained living in the affected area (13).

## Materials and Methods

### Study Design

This study was designed as a cohort study at three time points, using the 3-year follow-up dataset from the Mental Health and Lifestyle Survey (14). This survey is one of the detailed surveys of the Fukushima Health Management Survey (5). The target areas of the Mental Health and Lifestyle Survey included 13 municipalities: Hirono, Naraha, Tomioka, Kawauchi, Okuma, Futaba, Namie, Katsurao, Iitate, Minamisoma, Tamura, Kawamata, and highly affected areas in Date. These municipalities were the nationally designated evacuation zones (as of April 2011) inside Fukushima Prefecture, because of their proximity to the FDNPP. The wave 1 and wave 2 assessments were each performed in January of 2012 and 2013. The wave 3 assessment was performed in February 2014. These assessments were conducted through postal mail 10, 22, and 35 months after the disaster. The research group did not conduct the first assessment 12 months after the disaster to avoid transient psychological effects related to anniversary reactions. The comprehensive protocol of this survey has been published elsewhere (5).

### Study Population

Study respondents were the caregivers of children born between April 2, 1998 and April 1, 2004, who were in the first to sixth grade of elementary school on March 11, 2011, and living in one of the 13 municipalities that were the target areas of the Mental Health and Lifestyle Survey.

We used data for respondents who answered all three assessments sent directly by post to individual residents. The total number of children in the target area was 8,282 and the response rate of the assessments was 90.1% (*n* = 7,463) at wave 1, 55.2% (*n* = 4,574) at wave 2, and 45.9% (*n* = 3,799) at wave 3. Therefore, the number of study participants of this study was 2,626, constituting 31.7% of the target respondents. Familial relationships of the respondents were 1) mother, 90.1% (*n* = 2,357); 2) father, 7.7% (*n* = 201); 3) grandparents, 1.2% (*n* = 31); and 4) others, 0.8% (*n* = 22). The family member who responded to the questionnaires was decided by the family on a voluntary basis.

### Sociodemographic Information

Sociodemographic information such as sex, age at the disaster, disaster types experienced, the number of experiences at the disaster, and place of residence at wave 1 were examined. The experience of the earthquake and tsunami was asked using the following questions: “Did you experience the earthquake?” and “Did you experience tsunami?” Regarding the experience of the nuclear plant accident, we used the question, “Did you hear the sound of the nuclear plant explosion?” because many survivors had experienced intense fear after hearing the hydrogen explosions, which occurred three times during the first day and the fourth day after the earthquake. We assumed that this experience had caused direct, sharply defined horror for the residents, in addition to the vague fear of invisible radiation. We added data regarding the cumulative number of experiences at the disaster, in accordance with the assumption that the psychological burdens depend on the cumulative number of experiences at the disaster. The number of experiences at the disaster was calculated as the total of three kinds (earthquake, tsunami, explosion of the nuclear plant) of experiences at the disaster, ranging from 0 to 3. For place of residence, it was previously reported that many residents in the target area experienced frequent relocations after the disaster (7). To avoid exploring the complex nature of changes in place of residence over time, we only used the data regarding whether the respondent lived within or outside Fukushima Prefecture at the time of the survey at wave 1. Place of residence was categorized into within Fukushima Prefecture or outside Fukushima Prefecture.

### Assessments

Children’s victimization was assessed using the criterion “Picked on or bullied by other children over the last six months,” extracted from the peer relationship subscale of the Strengths and Difficulties Questionnaire (SDQ)–Parents’ Version (15–17). The SDQ is a 25-item questionnaire used for identifying psychopathological problems in children. The Japanese version has been reported to exhibit adequate internal consistency (18, 19) and convergent validity (19). In the question regarding children’s victimization, we did not ask the respondent whether the victimization was disaster-related or not. However, because this question was a part of the dataset of the Mental Health and Lifestyle Survey, it is possible that some caregivers thought this question referred to nondisaster settings in the postdisaster context.

### Statistical Analysis

We conducted chi-square tests to compare the prevalence of children at risk of being bullied on the basis of sociodemographic characteristics and disaster-related variables. Residual analyses were conducted to identify the contribution of variables. Cramer’s V was used for calculating effect sizes (0.1 as small, 0.3 as medium, and 0.5 as large). Ordered logistic regression analysis was conducted for multivariate analysis. We conducted the analyses with three assessments (wave 1, wave 2, and wave 3) independently. The existence of parental recognition of bullying (0 = Not true, 1 = Somewhat true, 2 = Certainly true) was the dependent variable. The independent variables of this analysis were sex (1 = female, 0 = male), age at the disaster, experience of earthquake (1 = yes, 0 = no), experience of tsunami (1 = yes, 0 = no), experience of nuclear plant explosion (1 = yes, 0 = no), and place of residence (1 = Outside Fukushima Prefecture, 0 = within Fukushima Prefecture). A significance level of 0.05 was used in the two-sided tests. All statistical analyses were conducted using IBM SPSS Statistics for Windows, Version 21.0 (IBM Corp., Armonk, NY, USA).

## Results

### Sociodemographic Characteristics and Disaster-Related Variables

Sociodemographic characteristics and disaster-related variables are shown in **Table 1**. The mean age and the standard deviation of children at the disaster was 9.33 ± 1.7 years. While almost all children experienced an earthquake, only 12.3% had experienced a tsunami. Approximately 40% of children heard the nuclear plant explosion.

**Table 1:** Sociodemographic characteristics and disaster-related variables of the study children (*n* = 2,616).

	*n*	%
**Sex**		
Male	1,326	50.7
Female	1,290	49.3
**Age at time of disaster (years)**		
6	28	1.1
7	463	17.7
8	465	17.8
9	463	17.7
10	424	16.2
11	406	15.5
12	367	14.0
**Disaster types experienced**		
Earthquake	2,590	99.0
Tsunami	321	12.3
Nuclear plant explosion	1,059	40.5
**Number of experiences at the disaster**		
0	11	0.4
1	1,426	54.5
2	981	37.5
3	194	7.4
Data missing	4	0.2
**Place of residence (current address) at wave 1**		
Within Fukushima Prefecture	2,087	79.8
Outside Fukushima Prefecture	518	19.8

### Parental Recognition of Bullying Victimization

**Table 2** shows the percentages of parental recognition of bullying victimization. Across the 3 years, around 80% responded “not true,” 15% responded “somewhat true,” and 5% responded “certainly true.”

**Table 2:** Percentages of parental recognition of bullying victimization, by waves.

	Wave 1 (2012)	Wave 2 (2013)	Wave 3 (2014)
Not true, *n* (%)	2,043 (78.1)	2,090 (79.9)	2,130 (81.4)
Somewhat true, *n* (%)	412 (15.7)	394 (15.1)	369 (14.1)
Certainly true, *n* (%)	154 (5.9)	124 (4.7)	103 (3.9)
Data missing, *n* (%)	7 (0.3)	8 (0.3)	14 (0.5)
Total, *n*	2,616	2,616	2,616

### Comparison by Sociodemographic Characteristics and Disaster-Related Variables

Comparisons of parental recognition of bullying victimization by sociodemographic characteristics and disaster-related variables are shown in **Table 3**. Chi-square tests revealed significant differences in parental recognition of bullying victimization for all independent variables, except age at the time of the disaster. Cramer’s V for all chi-square analyses were categorized into “small” effect sizes (0.11 or smaller).

**Table 3:** Comparison of parental recognition of bullying victimization, by sociodemographic characteristics and disaster-related variables, by waves.

	Wave 1 (2012), *n* (%)				
	Not true	Somewhat true	Certainly true	Chi-square	Cramer’s V
**Sex**					
Male	978 (73.9)	244 (18.4)	102 (7.7)	33.4**	0.11
Female	1,065 (82.9)	168 (13.1)	52 (4.0)		
**Age at the disaster**					
6	18 (64.3)	9 (32.1)	1 (3.6)	11.0	0.05
7	358 (77.3)	75 (16.2)	30 (6.5)		
8	360 (77.4)	80 (17.2)	25 (5.4)		
9	355 (76.8)	76 (16.5)	31 (6.7)		
10	332 (78.5)	64 (15.1)	27 (6.4)		
11	325 (80.4)	56 (13.9)	23 (5.7)		
12	295 (81.0)	52 (14.3)	17 (4.7)		
**Experience of earthquake**					
Yes	2,025 (78.4)	409 (15.8)	149 (5.8)	6.1*	0.05
No	15 (68.2)	3 (13.6)	4 (18.2)		
**Experience of tsunami**					
Yes	238 (74.4)	54 (16.9)	28 (8.8)	6.1*	0.05
No	1,802 (78.9)	358 (15.7)	125 (5.5)		
**Experience of nuclear plant explosion**					
Yes	789 (74.8)	192 (18.2)	74 (7.0)	13.1**	0.07
No	1,251 (80.7)	220 (14.2)	79 (5.1)		
**Number of experiences at the disaster**					
0	8 (72.7)	2 (18.2)	1 (9.1)	22.8**	0.09
1	1,156 (81.2)	194 (13.6)	73 (5.1)		
2	732 (74.8)	187 (19.1)	59 (6.0)		
3	144 (74.6)	29 (15.0)	20 (10.4)		
**Place of residence (current address) at survey at wave 1**					
Within Fukushima Prefecture	1,647 (79.1)	316 (15.2)	118 (5.7)	3.1	0.03
Outside Fukushima Prefecture	391 (75.6)	93 (18.0)	33 (6.4)		
	Wave 2 (2013), *n* (%)				
	Not true	Somewhat true	Certainly true	Chi-square	Cramer’s V
**Sex**					
Male	1,011 (76.4)	236 (17.8)	76 (5.7)	23.4**	0.10
Female	1,079 (84.0)	158 (12.3)	48 (3.7)		
**Age at the disaster**					
6	24 (85.7)	3 (10.7)	1 (3.6)	8.7	0.04
7	364 (78.8)	78 (16.9)	20 (4.3)		
8	374 (80.6)	64 (13.8)	26 (5.6)		
9	358 (77.7)	83 (18.0)	20 (4.3)		
10	342 (80.7)	64 (15.1)	18 (4.2)		
11	330 (81.9)	54 (13.4)	19 (4.7)		
12	298 (81.4)	48 (13.1)	20 (5.5)		
**Experience of earthquake**					
Yes	2,075 (80.3)	387 (15.0)	121 (4.7)	6.0	0.05
No	13 (61.9)	5 (23.8)	3 (14.3)		
**Experience of tsunami**					
Yes	251 (78.2)	44 (13.7)	26 (8.1)	9.2*	0.06
No	1,837 (80.5)	348 (15.2)	98 (4.3)		
**Experience of nuclear plant explosion**					
Yes	828 (78.3)	174 (16.5)	55 (5.2)	3.8	0.04
No	1,260 (81.4)	218 (14.1)	69 (4.5)		
**Number of experiences at the disaster**					
0	5 (50.0)	4 (40.0)	1 (10.0)	17.8**	0.08
1	1,163 (81.8)	197 (13.9)	61 (4.3)		
2	769 (78.5)	165 (16.9)	45 (4.6)		
3	151 (77.8)	26 (13.4)	17 (8.8)		
**Place of residence (current address) at wave 1**					
Within Fukushima Prefecture	1,691 (81.3)	290 (13.9)	100 (4.8)	10.3**	0.06
Outside Fukushima Prefecture	391 (75.8)	101 (19.6)	24 (4.7)		
	Wave 3 (2014), *n* (%)				
	Not true	Somewhat true	Certainly true	Chi-square	Cramer’s V
**Sex**					
Male	1,027 (77.9)	230 (17.5)	61 (4.6)	28.2**	0.10
Female	1,103 (85.9)	139 (10.8)	42 (3.3)		
**Age at the disaster**					
6	24 (85.7)	2 (7.1)	2 (7.1)	19.0	0.06
7	366 (79.0)	79 (17.1)	18 (3.9)		
8	376 (81.2)	63 (13.6)	24 (5.2)		
9	375 (81.2)	74 (16.0)	13 (2.8)		
10	337 (80.2)	67 (16.0)	16 (3.8)		
11	335 (83.3)	49 (12.2)	18 (4.5)		
12	317 (87.1)	35 (9.6)	12 (3.3)		
**Experience of earthquake**					
Yes	2,110 (81.9)	365 (14.2)	101 (3.9)	0.03	<0.01
No	18 (81.8)	3 (13.6)	1 (4.5)		
**Experience of tsunami**					
Yes	252 (78.5)	49 (15.3)	20 (6.2)	5.8	0.05
No	1,876 (82.4)	319 (14.0)	82 (3.6)		
**Experience of nuclear plant explosion**					
Yes	856 (81.2)	154 (14.6)	44 (4.2)	0.6	0.02
No	1,272 (82.4)	214 (13.9)	58 (3.8)		
**Number of experiences at the disaster**					
0	9 (81.8)	2 (18.2)	0 (0.0)	5.0	0.04
1	1,177 (83.1)	190 (13.4)	50 (3.5)		
2	785 (80.4)	150 (15.4)	41 (4.2)		
3	157 (80.9)	26 (13.4)	11 (5.7)		
**Place of residence (current address) at wave 1**					
Within Fukushima Prefecture	1,717 (82.7)	285 (13.7)	74 (3.6)	5.1	0.05
Outside Fukushima Prefecture	406 (78.8)	82 (15.9)	27 (5.2)		

*p < 0.05, **p < 0.01.

Across the three assessments, there were significant differences between boys and girls. Residual analysis revealed that the percentage of “somewhat true” and “certainly true” responses for boys were significantly higher than that for girls (|*r*| = 3.8 and 4.0, *p* < 0.01). There were no significant differences on age at the disaster.

With respect to disaster-related variables, there were significant differences in experiences for all types of disaster (earthquake, tsunami, nuclear plant explosion) at wave 1. However, only the experience of tsunami showed significant differences at wave 2, and none of the types showed significant differences at wave 3. Residual analysis revealed that the percentage of “certainly true” responses for children who had experienced tsunami at wave 1 was significantly higher than that for children who had not experienced tsunami (|*r*| = 2.3, *p* < 0.05). The percentages of “somewhat true” and “certainly true” responses for children who had experienced the nuclear plant explosion were significantly higher than those for children who had not experienced the nuclear plant explosion at wave 1 (|*r*| = 2.8 and 2.0, *p* < 0.01 and *p* < 0.05). The cumulative number of experiences showed significant differences at waves 1 and 2. At wave 1, the percentage of “somewhat true” responses for two experiences was significantly higher than that for other numbers of experiences (|*r*| = 3.6, *p* < 0.01), and the percentage of “certainly true” responses for three experiences was significantly higher than that for other numbers of experiences (|*r*| = 2.8, *p* < 0.01). At wave 2, a similar trend was observed, with the results revealing that the percentage of “somewhat true” responses for two experiences was significantly higher than other numbers of experiences (|*r*| = 2.0, *p* < 0.05), and the percentage of “certainly true” responses for three experiences was significantly higher than other numbers of experiences (|*r*| = 2.7, *p* < 0.01). Although there was no difference in the percentages according to the place of residence at waves 1 and 3, a significant difference was observed at wave 2. The Cramer’s V for the analyses were 0.03 at wave 1, 0.06 at wave 2, and 0.05 at wave 3. Residual analyses revealed that the percentage of “somewhat true” responses for children who lived outside the Fukushima Prefecture was significantly higher than that for children who lived within the Fukushima Prefecture (|*r*| = 3.2, *p* < 0.01).

### Multivariate Ordered Logistic Regression Analysis for Parental Recognition of Bullying Victimization

The results of the ordered logistic regression analysis for parental recognition of bullying victimization, by sociodemographic characteristics and disaster-related variables, by year, are shown in **Table 4**. Being male was significantly associated with the parental recognition of bullying victimization at wave 1 and wave 3. At wave 1, experiencing the nuclear plant explosion was significantly associated with parental recognition of bullying victimization. Moreover, age at wave 3 was negatively associated with parental recognition of bullying victimization.

**Table 4:** Ordered logistic regression analysis for parental recognition of bullying victimization.

	Wave 1 (2012)				Wave 2 (2013)				Wave 3 (2014)			
Independent variables	B	Wald	p	95% CI	B	Wald	p	95%CI	B	Wald	p	95% CI
Sex (1 = Female, 0 = Male)	−0.55	32.22	<0.01	[−0.75, −0.36]	−0.36	3.68	0.06	[−0.73, 0.01]	−0.38	5.73	0.02	[−0.69, −0.07]
Age at the disaster	−0.05	3.11	0.08	[−0.11, 0.01]	0.10	0.32	0.57	[−0.24, 0.44]	−0.18	3.84	0.05	[−0.36, 0.00]
Experience of earthquake (1 = Yes, 0 = No)	−0.74	2.77	0.10	[−1.62, 0.13]	0.06	0.01	0.92	[−1.11, 1.23]	−0.64	3.59	0.06	[−1.30, 0.02]
Experience of tsunami (1 = Yes, 0 = No)	0.18	1.56	0.21	[−0.10, 0.45]	0.27	1.12	0.29	[−0.23, 0.77]	−0.15	0.39	0.53	[−0.63, 0.33]
Experience of nuclear plant explosion (1 = Yes, 0 = No)	0.34	11.89	<0.01	[0.15, 0.53]	−0.38	0.93	0.33	[−1.15, 0.39]	0.35	1.33	0.25	[−0.24, 0.94]
Place of residence (1 = Outside Fukushima Prefecture, 0 = Within Fukushima Prefecture)	0.14	1.42	0.23	[−0.09, 0.37]	0.18	0.57	0.45	[−0.29, 0.65]	0.04	0.04	0.85	[−0.35, 0.43]

## Discussion

In this study, we found that sex, age at the disaster, and experience of nuclear plant explosion significantly influenced the prevalence of parental recognition in bullying victimization at some point during the study period. Regarding time course, the impact of sex was observed at wave 1 and wave 3; the impact of age was observed at wave 3, whereas the impact of experience of nuclear plant explosion was observed at wave 1. Together with the results of the univariate analysis that the cumulative number of experiences at the disaster was significantly associated until the 2-year follow-up, the disaster-related variables were considered larger effect at the earliest stage. Because our study did not include a control group that was not affected by the disaster, our method using the cumulative number of experiences could be helpful for estimating the quantitative mental health effects of the Fukushima disaster, which is considered a complex disaster (3, 4).

In the current study, the percentage of “somewhat true” responses for children was approximately 15%, and the percentage of “certainly true” responses was approximately 4%–6%. This is in line with previous large-scale studies in nondisaster conditions (9, 10). The percentage of “certainly true” responses was higher than the prevalence among evacuated children from the Fukushima Prefecture, reported by the Ministry of Education, Culture, Sports, Science, and Technology of Japan (2).

Although we did not find significant differences across 3 years in the multivariate analysis, our finding of an increase in the impact of place of residence at wave 2 in the univariate analysis suggested the existence of a qualitative change according to a mid- or long-term relocation of families who were evacuated from Fukushima Prefecture to other prefectures. A 3-year follow-up study of people who were evacuated from Fukushima Prefecture reported that the causes of stressors changed from the damage of the earthquake itself to the circumstances of living in shelters over time (20). A small-scale study showed that 28 children who were evacuated from the affected area of the FDNPP accident and lived in temporary housing in the nonaffected area of the Fukushima Prefecture experienced more frequent bullying than 106 children living in their own houses in Fukushima Prefecture and 321 children living in a nonaffected area in the Saitama Prefecture (21). The authors suggested that changes in friendship and the limitation of playground space may have influenced the results. According to the relationship between bullying and relocations at the disaster, a previous study of the impact of Hurricane Katrina demonstrated that an influx of new students in schools around a disaster area likely increases overall rates of bullying and victimization, as young people struggle to establish or maintain dominance in changing peer groups (11), in accord with social dominance theory (22).

Because nuclear disasters have unique characteristics, such as being invisible and odorless, it is important to compare the impacts with those of other invisible disasters, such as infectious disease outbreak or toxic chemical exposure. For example, after a railroad chemical spill of the toxic pesticide metam sodium in the United States, the spill-affected residents had greater environmental concerns about chemicals in the environment (23). Although we did not examine the existence of social stigma directly, stigmatization may be associated with an increase in the impact of bullying victimization according to the place of residence. A study of adult evacuees living outside Fukushima Prefecture revealed that 44% of males and 54% of females avoided telling others that they were evacuees from Fukushima, and 50% of males and 55% of females had bad experiences in regard to being evacuees (20). It is likely that relocated children also have had bad experiences with respect to being evacuees. Social stigma was also reported after other environmental disasters. Following a radiological accident in Goiania, Brazil, hotels in other parts of Brazil refused to allow residents of Goiania to register and some airplane pilots refused to fly airplanes that residents of Goiania aboard (24). In another case, a recent cohort study assessing Ebola-related stigma in Liberia demonstrated the existence of stigmatization against survivors 1 year after recovery from Ebola virus disease (25).

The current finding of a higher prevalence of bullying among boys was in accord with previous studies (9, 26). However, some previous studies have reported that girls are more susceptible to bullying under nondisaster settings (10, 27, 28). A recent study in Korea, which showed a higher prevalence of the experience of being bullied in girls, speculated that boys report being victimized less frequently than girls in Korea when self-reporting rather than peer-reporting methods are used (28). In addition, a previous study reported that the types of bullying differed between girls and boys (29). Because the respondents in the current study were caregivers, they may have been more likely to notice physical victimization more than nonphysical victimization. Regarding the effect of age group, the current study did not reveal significant differences, in contrast to previous studies reporting that bullying victimization decreased by age under a nondisaster setting (30).

The present study had several limitations that should be taken into account. First, we were not able to demonstrate causal associations between sociodemographic characteristics/disaster-related variables and parental recognition of bullying victimization. Second, parent-completed questionnaires may be less accurate than clinician-administered diagnostic tools. Third, we did not include a comparison group recruited from areas not affected by the disaster. Fourth, the response rates at wave 2 and wave 3 were relatively low. Fifth, social stigmatization was not examined in the questionnaire. Finally, we included only one question for detecting children at risk of bullying victimization.

Despite these limitations, the current study demonstrated that being male, the cumulative number of experiences at the disaster, and the place of residence were factors that significantly influenced the parental recognition of bullying victimization among children after the Fukushima disaster. Our findings may be helpful for informing the development of community- and school-based mental health care for children, parents, and teachers. For example, trauma-informed care may be a promising intervention (31). Trauma-informed care includes the perspective of children’s psychological trauma, and is beginning to be implemented in Japan (31, 32). Future research, including more detailed questionnaires on bullying victimization, is needed to examine risk and resilience factors against bullying victimization in relation to nuclear disasters.

## Ethics Statement

This study was carried out in accordance with the recommendations of STROBE Statement with written informed consent from all subjects. All subjects gave written informed consent in accordance with the Declaration of Helsinki. The protocol was approved by the Ethics Review Committee of Fukushima Medical University (number 1316) and the Ethical Committee of Kurume University (number 15188).

## Author Contributions

MM and MO conceived the original idea for the study. MO performed the analyses of the data and drafted the manuscript. TO, SI, MH, YS, HY, SY, KK, and HO contributed critical comments. All authors approved the final version.

## Funding

This study was supported by the national “Health Fund for Children and Adults Affected by the Nuclear Incident.”

## Conflict of Interest Statement

The handling Editor declared a past coauthorship with one of the authors (MO).

The authors declare that the research was conducted in the absence of any commercial or financial relationships that could be construed as a potential conflict of interest.
